# Software for spatial analysis and visualisation of cluster randomised trials - a review of available tools

**DOI:** 10.1186/1745-6215-16-S2-P34

**Published:** 2015-11-16

**Authors:** Christopher Jarvis, Gian Luca Di Tanna, Neal Alexander, Karim Anaya-Izquierdo, James Carpenter

**Affiliations:** 1 London School of Hygiene and Tropical Medicine, London, UK; 2 University of Bath, Bath, UK; 3 MRC CTU at UCL, London, UK

## 

In this context we define spatial analysis to mean Statistical Inference which takes into account spatial data, including but not limited to spatial exploratory data analysis. Groups in a Cluster randomised trial (CRT) are often defined geographically, giving spatial structure to the data. Taking account of the spatial aspects in the design and analysis of such a trial is important as it may influence results. In addition, the hierarchical structure imposed by the design should be adjusted for in the analysis. Therefore selecting software for spatial analysis of CRTs necessitates the capacity to accommodate for both spatial and clustering effects.

The range of spatial software available is broad in focus and capabilities, with a plethora of options to choose from determining what tool to use is not necessarily straight forward. In the last decade due to rapid development of spatial methods in Statistical packages and the expansion of Statistical toolboxes in Geographical Information Systems there is substantial overlap in abilities.

This review provides a comparison of current software options offering spatial analytical and visualisation techniques. First giving a brief account of the numerous cartographic visualisation tools, then focusing on what differentiates the capabilities of the analytical software. In regards to the latter we'll give special attention to multi-level modelling in both a Frequentist and Bayesian framework. Therefore demonstrating the flexibility of the options now available for the Analysis of CRTs taking into account spatial aspects.

